# Human recombinant truncated RNASET2, devoid of RNase activity; A potential cancer therapeutic agent

**DOI:** 10.18632/oncotarget.2562

**Published:** 2014-12-04

**Authors:** Liron Nesiel-Nuttman, Betty Schwartz, Oded Shoseyov

**Affiliations:** ^1^ The Robert H. Smith Institute of Plant Science and Genetics in Agriculture, The Robert H. Smith Faculty of Agriculture, Food and Environment, The Hebrew University of Jerusalem, Rehovot 76100, ISRAEL; ^2^ School of Nutritional Sciences Institute of Biochemistry, Food Science and Nutrition, The Robert H. Smith Faculty of Agriculture, Food and Environment, The Hebrew University of Jerusalem, Rehovot 76100, ISRAEL

**Keywords:** actin-binding, antitumorigenesis, antiangiogenesis, ribonuclease, trT2-50

## Abstract

Human RNASET2 has been implicated in antitumorigenic and antiangiogenic activities, independent of its ribonuclease capacities. We constructed a truncated version of human RNASET2, starting at E50 (trT2-50) and devoid of ribonuclease activity. trT2-50 maintained its ability to bind actin and to inhibit angiogenesis and tumorigenesis. trT2-50 binds to cell surface actin and formed a complex with actin *in vitro*. The antiangiogenic effect of this protein was demonstrated in human umbilical vein endothelial cells (HUVECs) by its ability to arrest tube formation on Matrigel, induced by angiogenic factors. Immunofluorescence staining of HUVECs showed nuclear and cytosolic RNASET2 protein that was no longer detectable inside the cell following trT2-50 treatment. This effect was associated with disruption of the intracellular actin network. trT2-50 co-localized with angiogenin, suggesting that both molecules bind (or compete) for similar cellular epitopes. Moreover, trT2-50 led to a significant inhibition of tumor development. Histological analysis demonstrated abundant necrotic tissue and a substantial loss of endothelial structure in trT2-50-treated tumors. Collectively, the present results indicate that trT2-50, a molecule engineered to be deficient of its catalytic activity, still maintained its actin binding and anticancer-related biological activities. We therefore suggest that trT2-50 may serve as a potential cancer therapeutic agent.

## INTRODUCTION

**Figure 1:**
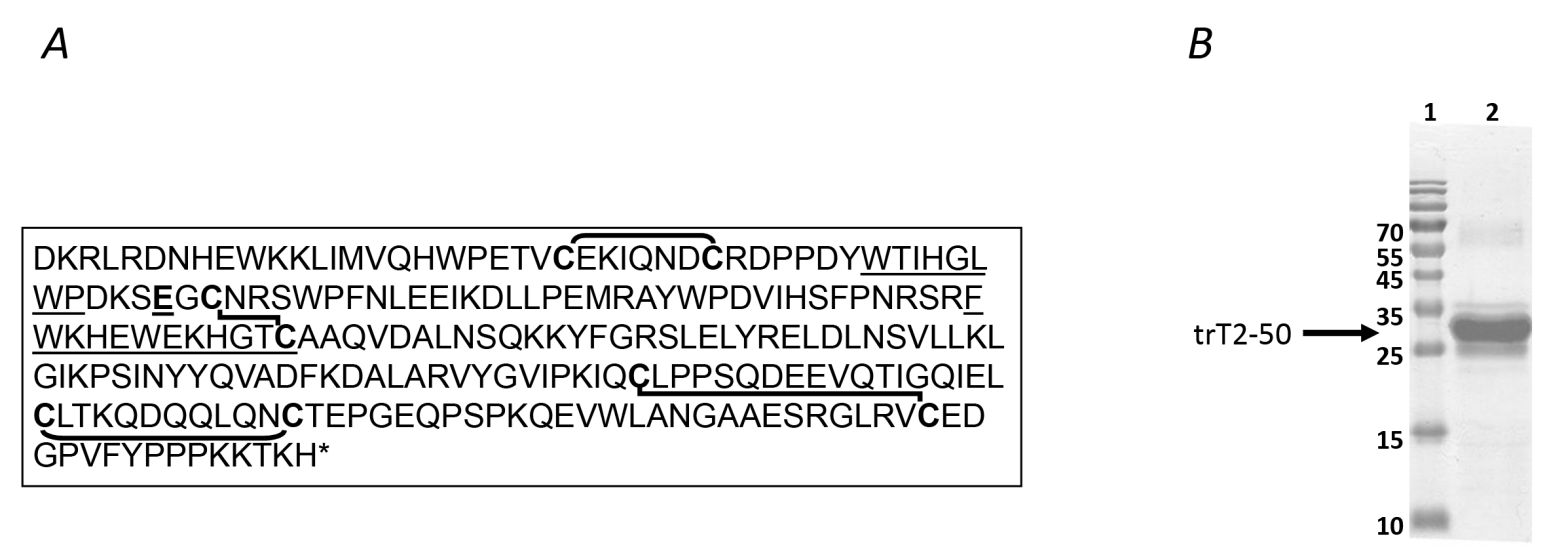
trT2-50 sequence and expression Human RNASET2 protein sequence **(A)**. The lines represent disulphide bridges between cysteine residues [11]. The RNase active sites is underlined. trT2-50 sequence begins at Glu 50 (E50) (Numbering is according to the synthetic gene optimized for E. coli expression, therefore refers to an additional methionine at the beginning of the protein sequence). Purified trT2-50 on a 12.5% SDS-PAGE **(B)**. lane 1, molecular markers. Lane 2, purified trT2-50.

Ribonucleases (RNases) are classified into the RNase T1, RNase A and RNase T2 families, according to their base specificity, structure, function, optimal pH and origin [1]. RNase A (e.g. onconases, bovine seminal RNase, α-sarcin) [2, 3 and ref. therein], RNase T1 (e.g. *Aspergillus orizae* origin) [4, 5] and RNase T2 (e.g. ACTIBIND and RNASET2) [6, 7, 8, 9] family members have been implicated in inhibition of tumorigenesis. Members of the RNase T2 family resemble the *Aspergillus orizae* T2-RNase (EC 3.1.27.1) and are widely distributed in living organisms, from viruses to mammals [10]. Two conserved active site (CAS) segments, CASI and CAS II, are of utmost importance for the ribonuclease activity of these enzymes [10, 11]. Animal and plant T2-RNases contain a total of eight cysteine residues, four of which are common to all T2-RNases and likely of fundamental importance to protein stability and/or function [1, 11]. Human RNASET2 disulfide bridges (Fig. 1A) have been characterized by Thorn *et al*., 2012 [12].

T2-RNases have been found in cellular domains where RNA is not readily available as a substrate, such as in extracellular domains or entrapped within vacuoles [13]. These findings have led researchers to speculate that these enzymes bear a biological function other than RNA processing. Our group was the first to isolate and characterize a T2-RNase from *Aspergillus niger* [14]. We established that this protein inhibits the elongation and alters the orientation of pollen tubes in plants by interfering with the intracellular actin network and therefore termed it ACTIBIND [14]. Cell motility and migration processes regulated by polymerization and depolymerization of intracellular actin are common activities to both pollen tube elongation and cancer cell motility, migration and proliferation [15, 16, 17], consequently ACTIBIND has been demonstrated to be antiangiogenic and anticarcinogenic [7, 8].

Human RNASET2 is a T_2_-RNase glycoprotein encoded by *RNASET2*, a recognized tumor suppressor gene located on chromosome 6 (6q27) [9, 18]. Gene deletion in this region has been associated with a variety of solid neoplasms, such as ovarian tumors [19], carcinomas of the breast [20], melanoma [21] and hematologic malignancies, such as non-Hodgkin's B-cell lymphoma [22] and acute lymphoblastic leukemia [23].

Amino acid sequence alignment between ACTIBIND (NCBI gi 71064123) and the putative human RNASET2-encoded protein showed 34% identity and 52% similarity. It was therefore expected that both human RNASET2 and ACTIBIND induce similar biological activities. Human recombinant RNASET2 (hrRNASET2) expressed in *Pichia pastoris* demonstrated actin-binding capacities [6]. Further *in vitro* assays and animal experiments demonstrated the antiangiogenic and antitumorigenic effects of hrRNASET2 [6]. Enzymatically inactive (EI) hrRNASET2 obtained after autoclaving at 120°C (120 kPa) for 30 minutes, still maintained its ability to bind actin and to inhibit colon cancer HT-29 colony formation, indicating that RNase activity is not a prerequisite for the antitumorigenic effect of hrRNASET2 [6]. Furthermore, Acquati *et al*., 2005 [24] reported that a double point mutation targeting the ribonuclease catalytic amino acids at the active site, led to a loss of RNase activity but did not affect its ability to inhibit tumor growth and metastasis. These findings were confirmed in an additional study conducted by our group in which both ACTIBIND and EI-ACTIBIND effectively arrested clonogenicity and tumor development in a rat cancer model [7].

The present study was designed to express a nonglycosylated form of hRNASET2 in a bacterial system. This was done due to the low protein expression yields in the P. pastoris expression system and the introduction of high levels of mannose glycosylation to the expressed protein. High levels of mannose glycosylation may constitute an obstacle toward future clinical translation [25]. In addition, the bacterial-expressed protein was also designed to corroborate that RNase activity is unnecessary for its antitumorigenic and antiangiogenic activities. For that purpose, a truncated form of hRNASET2 was expressed in *E. coli* (Figure 1A & 1B). This truncated protein starting at Glu50 is named hrtrRNASET2-50 (or in short, trT2-50) and designed to lack CASI. We demonstrate, herein, that RNase-inactive trT2-50 displays antitumorigenic and antiangiogenic activities in both *in vitro* and *in vivo* assays.

## RESULTS

### Affinity chromatography purification of trT2-50

Recombinant trT2-50, expressed in *E. coli*, was purified on a HisTrap affinity column and analyzed by SDS-PAGE (Figure 1B). A pure protein at the expected size of about 25 kDa (Figure 1B) was eluted in about 75 mM imidazol. trT2-50 bands were sent to LC-MS/MS analysis at the Smoler Proteomic Research Center (Biology Department, Technion - Israel Institute of Technology) for protein verification. The results confirmed that the proteins are truncated forms of the hRNASET2 based on the E.Coli-Human nr sequence database. The protein was demonstrated to be devoid of RNase activity (data not shown). Following protein purification and refolding, the protein was lyophilized and the yield was ~15 mg/100 ml growth medium.

### trT2-50 binds endothelial cell surface actin

**Figure 2:**
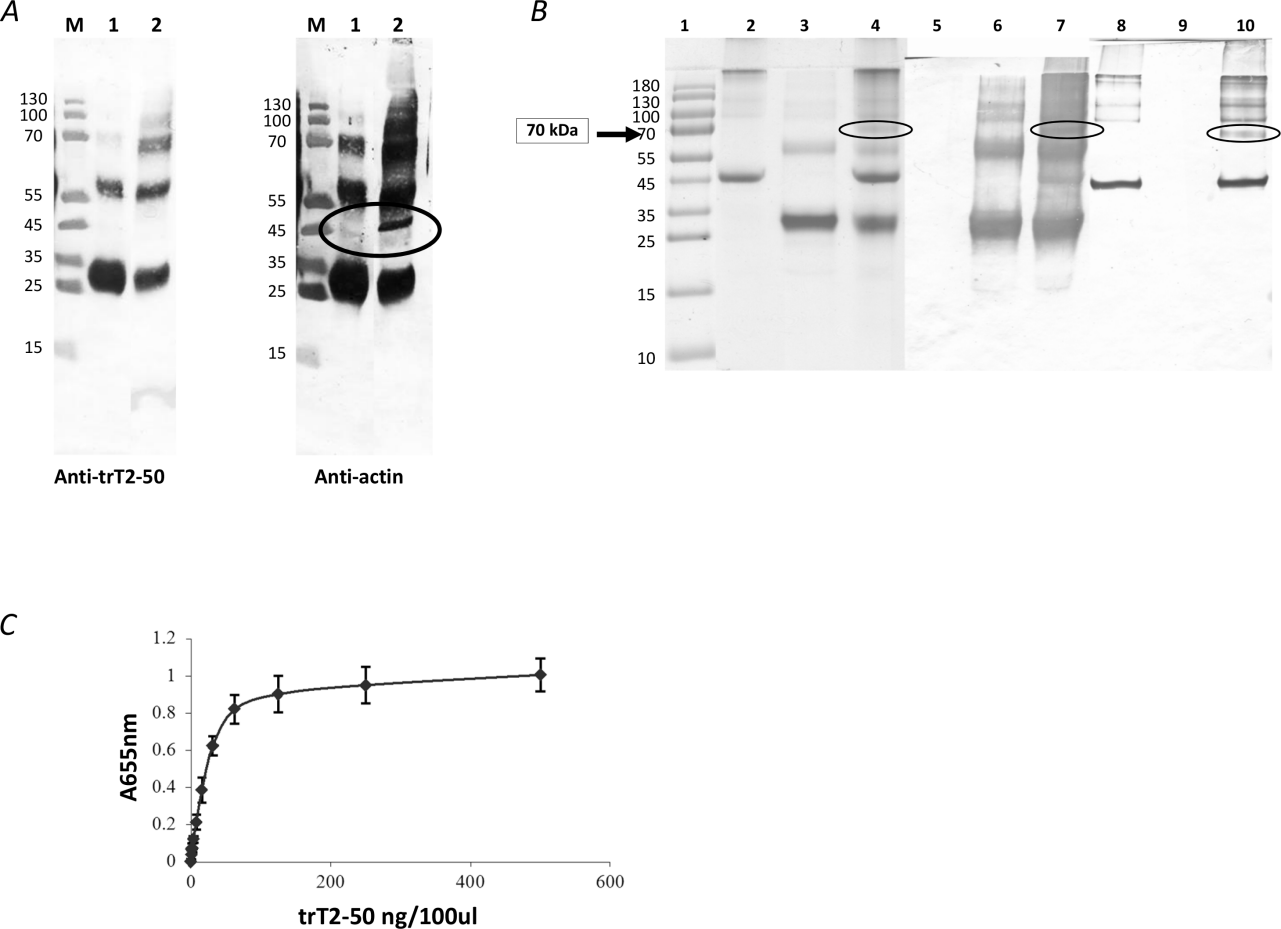
trT2-50 binds actin trT2-50 binds cell surface actin **(A)**. M, molecular markers. Lane 1, eluted fraction: his tag-trT2-50 was immobilized to Nickel beads. Supernatant solution of heparan-treated confluent starved HUVEC was added and incubated in rotation. Bounded fractions were eluted using 0.5M imidazol. Lane 2, supernatant solution of heparan-treated confluent starved HUVEC incubated with trT2-50 (without further purification). trT2-50 binds actin *in vitro* **(B)**. trT2-50 and actin form a complex of 70kDa in solution. Actin (10 μg) was incubated with 20 μg trT2-50 in Buffer G for 30 min at room temperature. EDC was then added and incubated for another 30 min. The reaction was then terminated and the cross-linked complex was analyzed by 10% SDS-PAGE stained with Coomassie blue or further analyzed by Western blot analysis upon incubation with anti-trT2-50 or anti-actin antibodies. Lane 1, molecular markers. Lanes 2-4, Coomassie blue staining: actin, trT2-50 and complex formation, respectively. Lanes 5-7, Western blot analysis using anti-trT2-50: actin, trT2-50 and complex formation, respectively. Lanes 8-10, Western blot analysis using anti- actin: trT2-50, actin and complex formation, respectively. trT2-50 binds actin on solid phase (ELISA) **(C)**. trT2-50 was added to immobilized actin at increasing concentrations from 0 to 500 ng/100μl/well, and then reacted with rabbit anti-trT2-50 and goat anti rabbit IgG-HRP. The amount of bound trT2-50 was quantified by measuring the optical density at 655 nm.

The dissociability of trT2-50-binding protein from HUVE cells was examined by treating the cells with heparan sulfate, a procedure sometimes used for release of cell-surface proteins [33]. Supernatant of heparan-treated confluent starved HUVEC was incubated with trT2-50 and was analyzed by Western Blot with anti-trT2-50 showing trT2-50 (monomer, and spontaneous dimers and trimmers) and with anti-actin showing a 42kDa protein which indicates that actin is present in the fraction of the supernatant of heparan-treated cells (Figure 2A, lane 2). Supernatant of heparan-treated confluent starved HUVEC was added and incubated in rotation to his tag-trT2-50 immobilized to Nickel beads. Bounded fractions were eluted using 0.5M imidazole and analyzed by Western Blot with anti-trT2-50 showing trT2-50 (monomer, and spontaneous dimers and trimmers) and with anti-actin showing the same 42kDa protein indicates that surface actin bound to trT2-50 (Figure 2A, lane 1).

### trT2-50 binds actin *in vitro*

Binding of trT2-50 to actin was analyzed by an in-solution cross-linking assay followed by gel electrophoresis and by solid-phase actin binding assay. trT2-50 and actin formed complexes *in vitro*, as evidenced by the 70 kDa band with Coomassie blue staining (Figure 2B, lane 4). The actin-trT2-50 complex was validated by blot analysis with anti-trT2-50 and anti-actin antibodies (Figure 2B, lanes 7 and 10, respectively). Solid phase analysis also showed that trT2-50 binds actin in a concentration-dependent manner with a ratio of ~1 : 2 (Figure 2C) and with a binding affinity of 15.7 × 10^−9^ M.

### trT2-50 inhibits HUVEC tube formation on Matrigel

**Figure 3:**
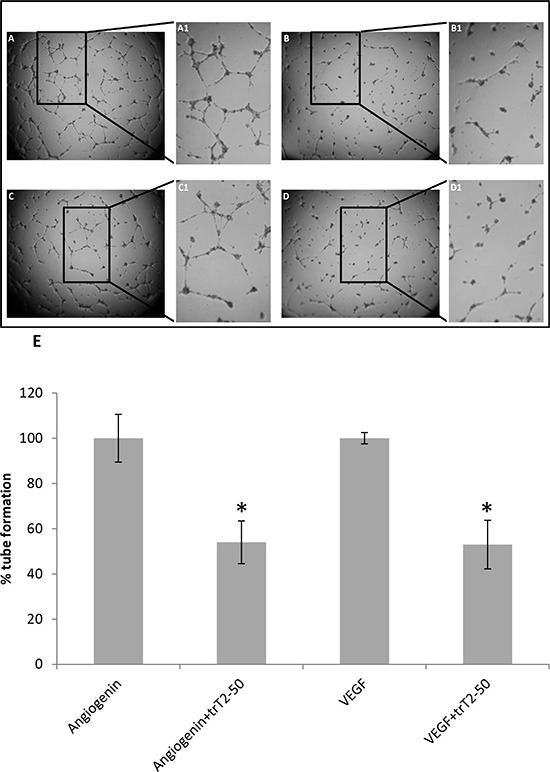
trT2-50 inhibits angiogenin and VEGF-induced HUVEC tube formation Freshly isolated HUVECs were plated in a 96-well plate previously coated with Matrigel. Cells were treated with either trT2-50 (2μM) **(B and D)** or PBS (control) **(A and C)**, in addition to angiogenin **(A and B)** or VEGF **(C and D)** (1 μg/ml each). After 8 h of incubation, at 37°C, the plates were photographed. Marked areas are digitally focused by 2-fold (A1-D1). Tube formation was assessed using Image J and the results are represented as percent of control **(E)**. trT2-50 had a significant inhibitory effect on tube formation (*P < 0.01).

The antiangiogenic effect of trT2-50 was assessed by HUVEC tube formation on Matrigel (Figure 3A, B, C, D). We observed that 2 μM trT2-50 treatment led to statistically significant ~50% inhibition (*P* < 0.01) angiogenin- and VEGF –induced tube formation compared to Control (Figure 3E).

### Nuclear and cytosolic protein is exported from the cell in the presence of trT2-50

**Figure 4:**
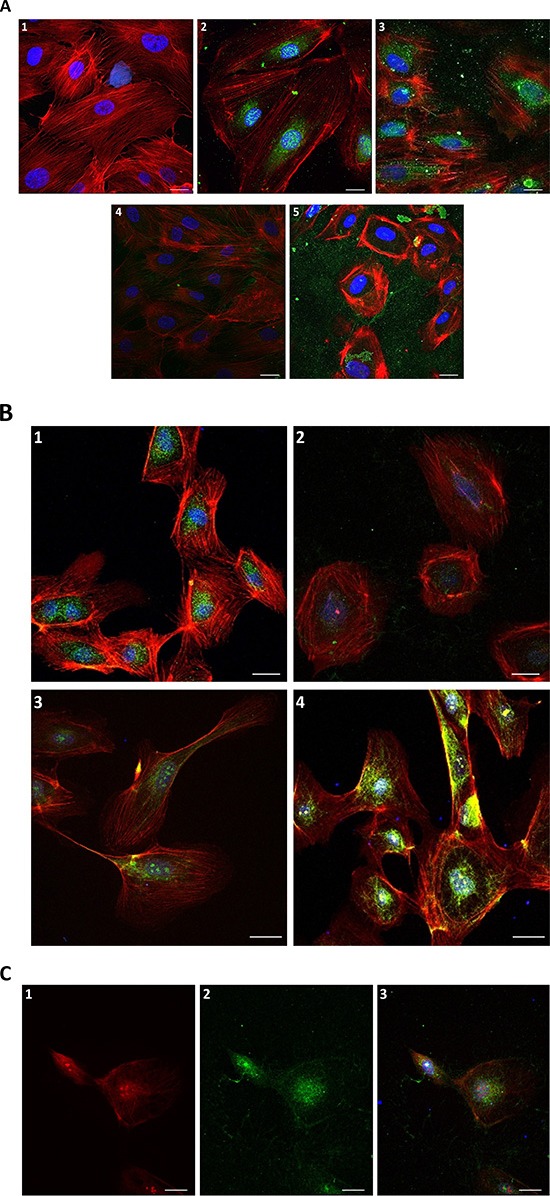
Immunofluorescence of HUVECs in the presence of trT2-50 and angiogenin Nuclear and cytosolic protein is exported from the cell in the presence of trT2-50 **(A)**: 1. Blank - HUVECs staining for actin (red) and for the nucleus (blue). 2. Control with anti-trT2-50 antibody – endogenous protein is located to the nucleus and the cytoplasm surrounding the nucleus (green). 3. Anti trT2-50 antibody, 10 min incubation with trT2-50 - endogenous protein disappears from the nucleus. 4. Anti trT2-50 antibody, 1 h incubation with trT2-50 - most of the endogenous protein disappears from the cell; minor part of the green signal was observed on the actin fibers. 5. Anti trT2-50 antibody, 48 h incubation with trT2-50 – green signal is observed merely outside the cell, the cells become rounded and loss their actin network structure. trT2-50 and angiogenin in HUVEC **(B)**: 1. Control with anti-hRNASET2 antibody is similar to that with anti-trT2-50 antibody – endogenous protein is located to the nucleus and the cytoplasm surrounding the nucleus (green). 2. Anti hRNASET2 antibody, 24 h incubation with trT2-50 – most of the endogenous protein disappears from the cell. 3. Control with anti-angiogenin antibody – endogenous angiogenin is located mainly in the nucleus and on the actin fibers (green). 4. Anti angiogenin antibody, 24 h incubation with angiogenin – same localization of angiogenin is observed in comparison with control, the signal was strengthened in the addition of external angiogenin (green). trT2-50 and angiogenin co-localize **(C)**. 24 h incubation with angiogenin and trT2-50: 1. Anti angiogenin antibody - angiogenin is located mainly in the nucleus, the cytoplasm surrounding the nucleus and on the actin fibers (red). 2. Anti hRNASET2 antibody - trT2-50 and the endogenous protein are located mainly in the nucleus, the cytoplasm surrounding the nucleus and some on the actin fibers (green). 3. The combine of 1 and 2, proteins generally co-localize. Scale bar = 20μm.

**Figure 5:**
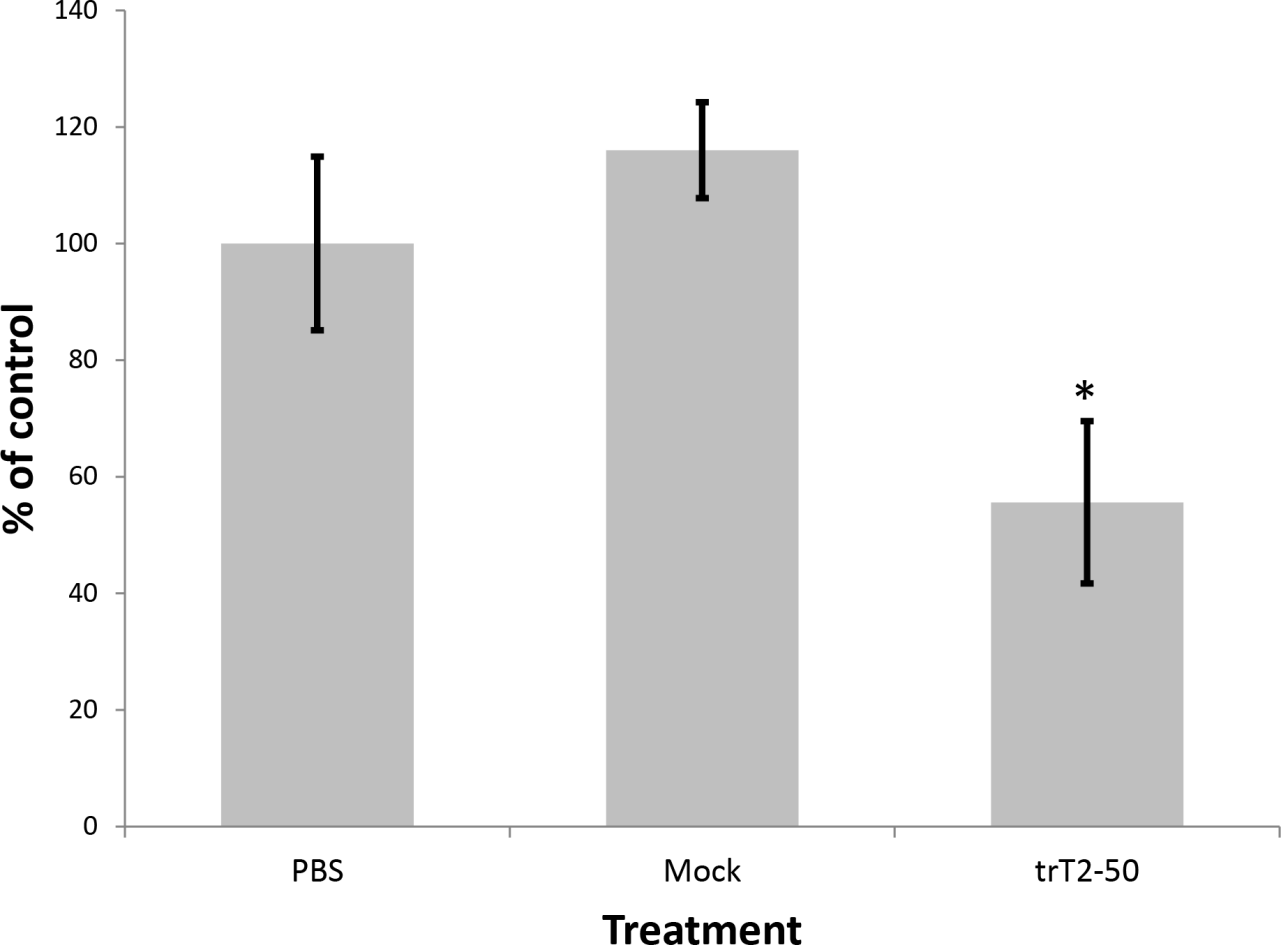
trT2-50 inhibits clonogenicity in colon cancer HT29 cells After 48 h incubation with trT2-50, control (PBS) or mock, cells were seeded in 96-well plates. The number of colonies after 5 days of treatment was counted using light microscope. Results are represented as percent of control (*P < 0.01)

In order to assess putative mechanisms of action by which trT2-50 inhibits tube formation in HUVE cells and therefore angiogenesis, we performed immunofluorescence staining studies and analyzed the localization of trT2-50 (10μM) when HUVECs were exposed to the recombinant protein *in vitro* for different time periods (Figure 4A; 3, 4, 5). Control samples were not treated with trT2-50, but were incubated for the same time periods with PBS (Figure 4A; 2). Exposure of HUVE cells to trT2-50 triggered a response mechanism by which a nuclear RNASET2 protein was time-dependently exported from the nucleus to the cytosol and subsequently outside the cell. Following 1 h of incubation with trT2-50 most of the endogenous protein disappears from the cell and only minor signals are observed on the actin fibers. Following 24 h (data not shown) and 48 h (Figure 4A; 5) of incubation with trT2-50, signal of either trT2-50 or endogenous hRNASET2 is observed only beyond the cell boundaries, thereafter the cells became rounded and lose their normal actin network structure. The activities described above became more accentuated with longer exposure to trT2-50. We suggest that these cellular effects are closely associated to the inhibitory effect that trT2-50 exerts on tube formation of HUVE cells and therefore on angiogenesis.

### trT2-50 co-localizes with angiogenin

The immunofluorescent micrographs presented in Figure 4B and 4C clearly demonstrate that the trT2-50 protein co-localizes with angiogenin. Following 24 h treatment with trT2-50 the cellular signal of angiogenin was significantly reduced (Figure 4C1 and 4C3) when compared to cells treated with only angiogenin (Figure 4B4).

Additionally, exposure of HUVE cells to angiogenin for 24 h induced a delay in both, the nuclear and the cytosolic protein disappearance (Figure 4C2 and 4C3). We therefore suggest that both molecules bind (or compete) for similar cellular epitopes as previously reported for ACTIBIND [8].

### trT2-50 inhibits clonogenecity of tumor cells

The antitumor effect of trT2-50 was assessed by its ability to inhibit colony formation of HT-29 colon cancer cells as compared with control (PBS) and mock. Cells were seeded in 96-well plates in the presence or absence of 1 μM trT2-50, PBS (control) or mock. The results demonstrate that trT2-50 significantly attenuated colonies formation by approximately 50% (Figure 5) (*P* < 0.01, *N* = 5 for each treatment).

### trT2-50 inhibits tumor progression *in vivo*

**Figure 6:**
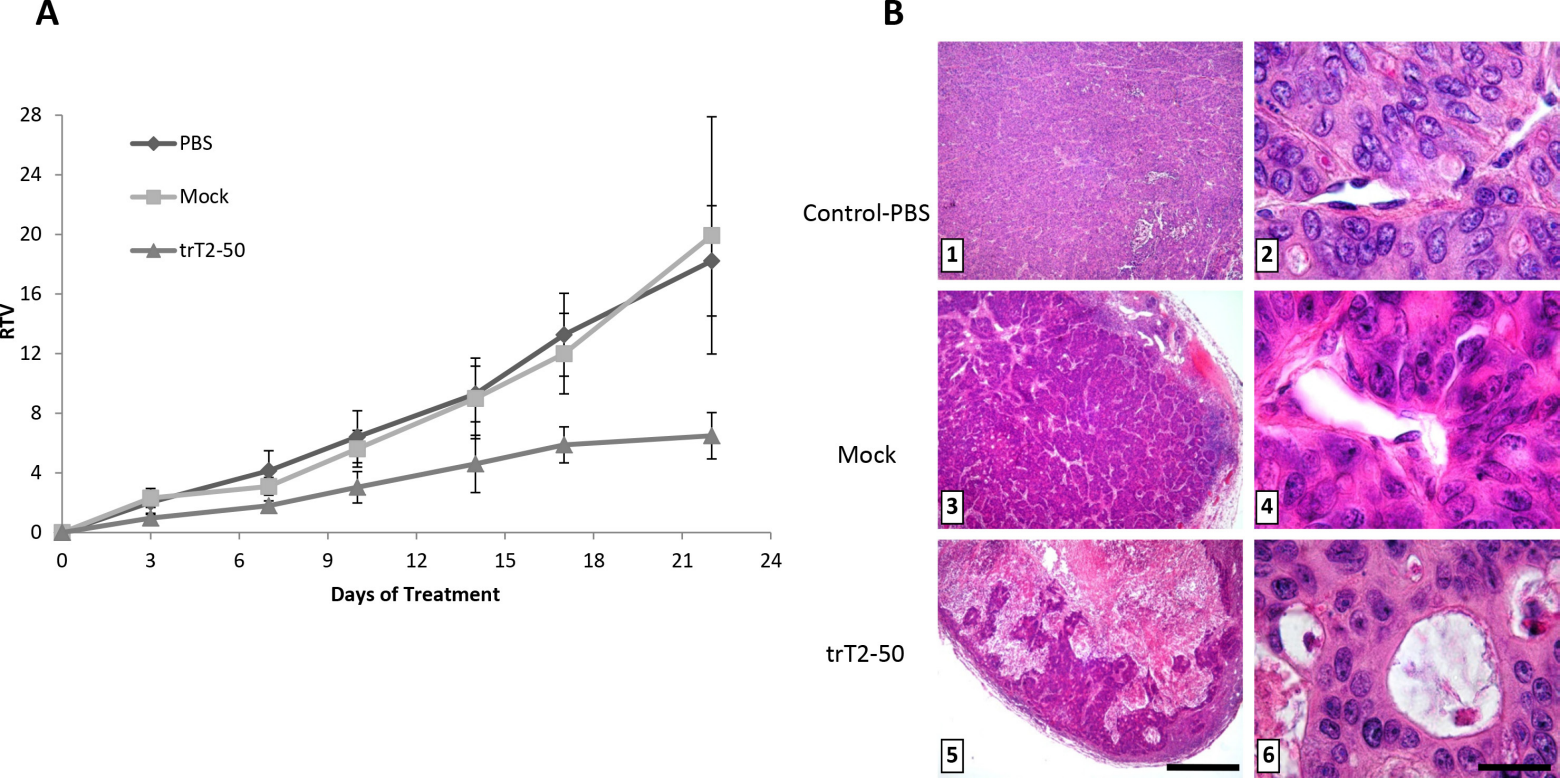
trT2-50 inhibits tumor progression and angiogenesis *in vivo* trT2-50 inhibits tumor progression *in vivo* **(A)**. Viable human colon cancer HT-29 cells (0.5 × 10^6^ cells/100 μl) were subcutaneously injected into the left hip of athymic mice. When the tumor diameter reached 5-7 mm, trT2-50 (5 mg/kg), mock (equivalent amount in PBS) or PBS alone (control) were injected into the tail vein, every other day, to a total of nine injections. Tumors were measured twice a week; the results are presented as the mean relative tumor volume (RTV). Each bar represents the standard error of the mean. *N* = 5. Photomicrographs show H&E tumors sections from control and treated mice. Treatment with trT2-50 proves that the tumor cells are concentrated in clusters surrounded by extensive necrotic tissue (5) and detached from the endothelium (6). In contrast, in control and mock-treated tumors, cancer cell invasion was observed over an extensive area, which contained large blood vessels (1 and 3, respectively). Endothelial cells located next to tumors cells showed intact morphology (2 and 4, respectively) **(B)**. Scale bar = 375 mm (1, 3, 5); 15 mm (2, 4, 6).

The ability of trT2-50 to affect the growth rate of HT-29 human colon cancer cells was assessed in the xenograft athymic mouse model. Mice were treated with either trT2-50 (5 mg/kg), mock (equivalent amount in PBS) or PBS (100 μl), intravenously administered every other day, to a total of nine injections. As shown in Figure 6A, tumor sizes were significantly reduced in trT2-50-treated mice, in comparison to the control groups. Control mice (PBS or mock) developed tumors with a wide range of sizes (RTV = 12–28). In contrast, trT2-50 treated mice developed tumors with significantly lower sizes (RTV = 4–6) and size variability. Cumulatively, trT2-50 treatment led to a statistically significant 60% inhibition of the tumor size, when compared with the control groups. trT2-50 could inhibit tumor growth *in vivo* by binding tumor cells directly or by binding to tumor-associated endothelial cells. trT2-50 may therefore inhibit both the development of the tumor and the formation of blood vessels that provide the tumor cells with oxygen and other essential nutrients.

### trT2-50 inhibits angiogenesis *in vivo*

Xenografts experiments were terminated 21 days after the initial treatment. Mice were sacrificed and tumors were fixed and cross-sections (6–7 μm) were stained with H&E. Histologic analysis of these tumor sections demonstrated the effect of the different treatments on tumor blood vessels (Table 1, Figure 6B). In control (Figure 6B; A, B) and mock-treated mice (Figure 6B; C, D), HT-29 cancer cell invasion was observed over an extensive area, which contained numerous blood vessels. Endothelial cells located next to tumors cells showed intact morphology in all control sections (Figure 6B; B, D). In contrast, sections obtained from mice treated with trT2-50, contained tumor cells concentrated in clusters surrounded by extensive necrotic tissue (Figure 6B; E). Higher magnification showed that the tumor cells detached from the blood vessel and a substantial loss of endothelial structure was observed (Figure 6B; F). Analysis of blood vessel in the tumor median cross-sections indicated a significant inhibitory effect of trT2-50 on the total vessel area (numbers) and on relative tumor area (66% and 79% decreases, respectively, Table 1, *P* < 0.01) compared to control.

**Table 1:** trT2-50 inhibits the total vessel area and the relative tumor area compared to control and mock samples

	Control (*n* = 10)	Mock (*n* = 10)	trT2-50 (*n* = 16)
**Total vessel area (mm^2^)**	8,527^a^ ± 966	13,621^b^ ± 1,444	2,915^c^ ± 505

Mean ± SE, a-c results with different letters are significantly different (*P* < 0.01).

## DISCUSSION

In this study, we examined the actin-binding capacities and the antitumorigenic and antiangiogenic effects of trT2-50, a novel RNase- inactive truncated form of hRNASET2, expressed in and purified from *E. coli*. The *E. coli* system proved a suitable expression host for trT2-50, due to ease of handling, cost effectiveness and high yields [34].

We have previously reported that T2-RNases, in order to be biologically active should bind actin [6, 7, 8]. In line with our previous reports we demonstrate herein that the novel truncated protein also binds HUVEC surface actin and binds actin *in vitro* (Fig. 2A, B, C). trT2-50 inhibited tumorigenesis *in vitro* by significantly reducing the clonogenic ability of cancer cells (Fig. 5). The antiangiogenic effect of trT2-50 *in vitro* was demonstrated by the significant inhibitory activity exerted on angiogenin- and VEGF-induced HUVE cell tube formation (Fig. 3). We further showed that the endogenous RNASET2 protein, expressed in the nuclei and the cytoplasm of HUVECs, is exported beyond the cell boundaries following exposure to trT2-50 (Fig. 4A). We assume that this translocation allows RNASET2 to bind cell surface actin. As a result, after 48 hours of incubation with trT2-50 the cell shape is drastically affected and they became rounded while losing their actin network structure (Fig. 4A). We assume that this cellular effect induced by RNAseT2 is closely associated with the inhibitory effect of trT2-50 on cell motility.

Supporting the in *vitro* findings, the antitumorigenic and antiangiogenic effects of trT2-50 were also demonstrated *in vivo* in HT-29-derived xenograft model (Fig. 6). In athymic mice, trT2-50 injected into the tail vein, significantly inhibited viable human colon cancer HT-29-derived xenograft development (Fig. 6A). Furthermore, trT2-50 affected the histology of tumors and blood vessels (Fig. 6B) and inhibited angiogenesis *in vivo* (Table 1).

Regarding the structure of trT2-50, out of eight cysteine residues, forming four disulfide bonds in the full-length hRNASET2, trT2-50 contained only six residues. The first two disulphide bonds are common to all RNases of T2 family and hence might have a fundamental importance to maintain the ribonuclease active conformation [1, 10, 13]. The lack of the first disulphide bond in the truncated protein may affect the correct folding of the recombinant protein, however, its actin-binding capacity and biological activity was conserved. This biological activity is similar to that of the full-length RNASET2 [6]. In addition, trT2-50 is missing the CASI site that is essential for RNase activity, consequently leads to its inability to degrade RNA.

Over the years, RNases purified from multiple origins have drawn increasing attention from medical scientists due to their remarkable antitumor properties [35, 36, 37, 38, 39, 40]. Application of RNases toward diagnosis and treatment of diseases are suggested to be RNase-based mechanisms [2, 35]. Members of the RNase A family (e.g. onconase, BS-RNase) were found to drive their cytotoxic effect by their ability to bind certain cells, enter the cytosol, and degrade RNA, thereby inhibiting protein synthesis, leading to cell death [36, 41, 42]. Strategies, such as site-directed mutagenesis, multimerization, fusion to a targeting moiety and chemical modifications, improve the antitumor potency as well as the specificity of some RNases and reduce their side effects [40]. On the other hand, in members of the RNase T2 family, biological activity is not related to their ability to degrade RNA, as previously reported for ACTIBIND [7, 8] and for the full-length human RNASET2 [6]. These results are consistent with those reported by Acquati *et al* (2005) for human RNASET2, in which a double point mutation at the catalytic site did not suppress its anti-cancer effect [24]. In this report, we clearly demonstrate that the RNase activity is not necessary for the antitumorigenic and antiangiogenic effects of RNASET2, implying that RNases can simultaneously influence several functions in the tumor cells, unrelated to their RNASE activity.

In cancer cells, the structure of the actin network is directly related to their malignant potential [43]. Interference of actin cytoskeleton organization in both endothelial and vascular smooth muscle cells directly affects their migration capacity, thereby inhibiting angiogenesis [17, 44]. Furthermore, surface actin in endothelial cells has been demonstrated to serve as a receptor for angiogenin, plasminogen and tissue plasminogen activator, proteins which play central role in angiogenesis [27, 45, 46]. We have shown previously that ACTIBIND competes with angiogenein on actin binding [8] disrupted the intracellular cytoskeletal network and triggered F-actin accumulation towards the cell-membrane boundaries in HT-29 colon cancer cells [7]. The results were supported by previous studies in which antibodies against either angiogenin or actin delayed or prevented the formation of HT-29 tumors in athymic mice [47, 48]. As trT2-50 binds surface actin, we suggest that this truncated protein, resembles the activities exerted by ACTIBIND, interfere with the actin organization in both endothelial and cancer cells and therefore results in inhibition of angiogenesis and tumorigenesis. As migrating cells display higher levels of actin polymerization compared to stationary cells [49], they are anticipated to be more sensitive to trT2-50 treatment. Thus, the described trT2-50 is proposed to target cancer cells effectively.

In summary, the present findings demonstrate that trT2-50, a non-glycosylated version of the full-length human RNASET2 devoid of ribonuclease activity, still maintains its actin-binding activity and strongly inhibits tumor progression and angiogenesis. We suggest that this newly designed recombinant protein may serve as a potential anticancer-antiangiogenic therapeutic agent. This novel molecule contains a portion identical to that of the corresponding human protein; therefore, antigenicity is not anticipated. We have recently identified the actin binding sequence of hRNASET2 (manuscript in preparation). This may lead to plan shorter and more effective molecules than the native human RNASET2, to improve its anti-angiogenic and anti-carcinogenic effects in cancer therapy.

## MATERIALS AND METHODS

### Cloning of *trT2-50*

An *hRNASET2* synthetic gene (GENEART GmbH, Regensburg, Germany) was used as the DNA template for the construction of truncated human recombinant RNASET2 (trT2-50). *trT2-50* was constructed by PCR carried out with a Mastercycler gradient PCR (Eppendorf, Hamburg, Germany). The PCR mix contained: 10 ng DNA template, 5 μl 10X *Imax Taq* polymerase buffer, dNTP mix (0.2 mM of each nucleotide), 0.4 pmol of each primer (forward: 5′ CCA TGG GTG ACG ATG ATA AAG AAG GCT GTA ATC GTA GCT GGC CGT TC 3′ and reverse: 5′ GCG GCC GCA AGC TTG GAT CCT TAG 3′), 1 unit of *Imax Taq* DNA polymerase (iNtRON, Korea) and ddH_2_O to a final volume of 50 μl. Forward primers contained the sequence of the enterokinase (EK) recognition site, to enable the removal of the His tag attached to the N-terminus of the proteins, if desired. The amplified fragments were screened on an agarose/TBE gel. The relevant bands were isolated and purified using the Wizard SV Gel and PCR Clean-up system (Promega, Madison, WI). The purified fragments were ligated into the pGEM plasmid (T-A cloning, Promega), in a reaction carried out overnight, at 15^°^C, in rapid ligation buffer (Bio-Lab LTD, Jerusalem, Israel). The ligase was inactivated at 65^°^C for 10 min. The pGEM plasmid containing the relevant DNA fragments and the pHIS-Parallel3 vector (generously provided by Dr. Tsafi Danieli at the Protein Expression Facility, The Wolfson Center for Applied Structural Biology, The Hebrew University of Jerusalem) was cleaved with *Nco*I and *Bam*HI restriction enzymes (Fermentas AB, Vilnius, Lithuania). The DNA fragments were isolated on a 1% agarose gel and extracted using the Wizard SV Gel and PCR Clean-Up System (Promega). Isolated DNA fragments were ligated into the cleaved plasmids. Ligation was carried out at 15^°^C, overnight, in rapid ligation buffer (Fermentas AB) in the presence of 5U T4-ligase (Fermentas AB) and then inactivated at 65^°^C for 10 min.

### Transformation into *E. coli*

Competent *E. coli* cells were prepared as described in Ausubel *et al*. (1998) [26] and maintained at −70^°^C. The ligation product (8 μl) was transformed into competent *E. coli* DH5α by heat shock. Bacteria were then seeded on LB ampicillin contacting plates. For plasmid extraction, a single colony was selected and inoculated in 5 ml LB medium, at 37^°^C, in a rotary shaker (250 rpm). Plasmid DNA extraction was carried out with the Jetquick Plasmid Miniprep Spin Kit (Genomed Inc., Gmgh Lohne, Germany) and used to transform 60 μl *E. coli* BL21 (DE3) competent cells using heat shock. Bacteria were then seeded on LB ampicillin contacting plates. Plasmids were sequenced at the Center for Genomics Technologies, The Institute of Life Science, Hebrew University of Jerusalem, Israel.

### Protein expression

*E. coli* BL21(DE3) colonies containing the *trT2-50* gene in the pHIS-Parallel3 vector were cultured overnight (37^°^C and 250 rpm) in 10 ml LB medium. Culture samples (6 ml) were then transferred to 400 ml LB medium and grown to OD_600_ = 0.6-0.8. Protein expression was induced by addition of 1 mM isopropyl β-D-thiogalactopyranoside (IPTG) (Dushefa, Haarlem, The Netherlands) for 3 h at 37^°^C, 250 rpm. The cells were then harvested by centrifugation at 14,000 g for 10 min at room temperature. *E. coli* BL21(DE3) colonies containing an empty vector (mock) were grown in parallel as described above, and served as control.

### Cell lysis

After centrifugation, bacterial cell pellets of 100 ml cultures were resuspended in 25 ml lysis buffer containing 20 mM phosphate buffer, 8 M urea, 0.1 NaCl, 1 mM EDTA (pH 8) and 2 mg/ml complete protease inhibitor (Roche Diagnostics, Mannheim, Germany). The harvested cells were than stirred for 2 h at 4^°^C. The lysates were centrifuged at 14,000 g for 30 min at room temperature and the supernatant was filtered (Whatman^®^ FP30/0.2 μm, cellulose acetate filter).

### Protein purification

Filtrated bacterial lysate containing the recombinant proteins was loaded, in lysis buffer, onto a 1 ml HisTrap Ni-Sepharose affinity column (GE-Healthcare Bio Sciences AB, Uppsala, Sweden). Proteins were eluted with an imidazol gradient (5-500 mM), prepared in equilibration buffer containing 20 mM sodium phosphate (pH 8.0), 1 M NaCl, 8 M urea and 5 mM β-mercaptoethanol, at a flow rate of 1 ml/min using GE-Healthcare's ACTAprime plus FPLC system (GE-Healthcare Bio Sciences AB). The fractions collected from the peak (~75 mM imidazol) were analyzed by 12.5% SDS-PAGE followed by Coomassie R250 staining. Protein containing fractions were pooled for refolding.

### Protein refolding

The purified protein was refolded by dialysis against 20 mM Tris solution (pH 12.0). Dialysis solution was exchanged once per hour for four hours and then left overnight at room temperature. The same procedure was then used with 20 mM Tris solution (pH 10) and finally with 20 mM ammonium bicarbonate buffer (pH 8). The refolded protein was lyophilized and kept at 4^°^C.

### RNase activity assay

A zymogram for RNase activity was performed as modified from Roiz *et al*. 2000 [14]. Samples of 5 μl were placed over a plate that contained 10 ml of 0.1% yeast RNA and 0.8% agarose in 20 mM sodium acetate and were incubated at 37°C for 30 minutes. Then, the agarose plate was stained with 0.02% (weight/volume) toluidine blue in water. RNase activity was visualized as a bright area on a blue background.

### *In vitro* assays

#### Release of cell-surface proteins by heparan sulfate

HUVE cells obtained from Lonza Walkersville Inc. (Walkersville MD. Lot number 0000187910) were derived from human umbilical endothelial cells. The cells were cultured in endothelial growth media (EGM) supplemented with SingleQuots (EGM BulletKit CC-3124, Lonza).

Subconfluent starved-cell monolayers were washed three times with phosphate-buffered saline (PBS), and 0.4 ml of PBS containing 1 mg of heparan sulfate per ml was added. The cells were incubated at room temperature for 30 min with occasional shaking, and the released material was removed and clarified by centrifugation at 1500 rpm (700 × g) for 5 min [27].

### Purification of trT2-50 binding protein

trT2-50 was immobilized onto Ni-NTA Agarose beads (GE-Healthcare Bio Sciences). Supernatant solution of heparan-treated confluent starved HUVEC was added and incubated in rotation. Bounded fractions were eluted using 0.5M imidazol in equilibration buffer containing 20 mM sodium phosphate (pH 8.0) and 1 M NaCl.

The eluted fractions collected were analyzed by Western blot analysis using anti-trT2-50 antibody (whole serum, Anilab, Israel) or anti-actin antibody (A2066, Sigma-Aldrich). Membranes were then incubated with goat anti-rabbit antibody conjugated with alkaline phosphates (Sigma-Aldrich). Developing was performed with BCIP (5-Bromo-4-chloro-3-indolyl phosphate, Sigma-Aldrich) and NBT (Nitro Blue Tetrazolium, Sigma-Aldrich) substrates dissolved in DMF (N, N-dimethylformamide, Sigma-Aldrich) in developing buffer (1.5 M Tris pH 8.8, 6 mM MgCl_2_, 0.1 M NaCl) and incubated for ~10 min.

### In solution actin binding assay

Assay was performed as previously described [6]. Actin (10 μg) (Sigma-Aldrich, St. Louis, MO) was incubated with 20 μg trT2-50 in 15 μl Buffer G (2 mM Tris-HCl (pH 8.0), 0.2 mM CaCl_2_ and 0.2 mM ATP), for 30 min at room temperature. EDC (Sigma-Aldrich) was then added to a final concentration of 10 mM and incubated for another 30 min. The reaction was quenched with an equal volume of sample application buffer (SAB) and the cross-linked complex was analyzed by 10% SDS-PAGE, followed by Coommasie R250 staining or Western blot analysis using anti-trT2-50 antibody (whole serum, Anilab, Israel) or anti-actin antibody (Sigma-Aldrich). Membranes were then incubated with goat anti-rabbit antibody conjugated with alkaline phosphates (Sigma-Aldrich). Developing was performed with BCIP (5-Bromo-4-chloro-3-indolyl phosphate, Sigma-Aldrich) and NBT (Nitro Blue Tetrazolium, Sigma-Aldrich) substrates dissolved in DMF (N,N-dimethylformamide, Sigma-Aldrich) in developing buffer (1.5 M Tris pH 8.8, 6 mM MgCl_2_, 0.1 M NaCl) and incubated for ~10 min.

### Actin binding solid phase assay

96-well plates (MaxiSorp^®^ flat-bottom 96-well plate, Fisher Scientific Inc., Fair Lawn, NJ) were coated with 500 ng actin in 100 μl carbonate-bicarbonate buffer (pH 9.5) for 1 h at 37°C. The plate was washed once with TBS and then blocked with 3% BSA in 200 μl TBS buffer at 37°C, for 1 h. Wells were then washed once with 250 μl TBS. trT2-50 was added at 1:2 dilutions in 100 μl TBS, starting from 500 ng/well, incubated for 1 h at 37°C and then washed three times with TBS containing 0.1% Tween-20 (TBST). Each well was then treated with 100 μl rabbit anti-trT2-50 diluted 1:500 in TBS and incubated for 1 h at 37°C. The wells were washed three times with TBST and then incubated with 100 μl goat anti-rabbit IgG-HRP (Jackson ImmunoResearch, West Grove, PA) diluted 1:10,000 in TBS, for 1 h at 37°C. Wells were then washed twice with TBST, and once with TBS before 100 μl substrate (1-step Ultra TMB-ELISA, Pierce, Fisher Scientific Inc.) were added. Absorbance at 655 nm was measured 10 min thereafter using a Power Wave 200 Microplate Scanning Spectrophotometer (Bio-Tek Instrument, Winooski, VT). Affinity was evaluated by double reciprocal plot [28, 29].

### Human umbilical vein endothelial cell (HUVEC) angiogenesis assay

Freshly isolated HUVEC were maintained in endothelial cell growth medium (EGM) supplemented with SingleQuots (EGM BulletKit CC-3124, Lonza). They were then plated in a 96-well plate (14 × 10^3^ cells/well) previously coated with growth factor-depleted Matrigel (Becton-Dickinson, Bedford, MA) in M199 medium supplemented with 20% FCS, 1% glutamine, 1% antibiotic–antimycotic solution (Biological Industries) and 50 U/100 ml heparin (Biomedical Technologies Inc., Stoughton, MA). Cells were simultaneously treated with either trT2-50 (2 μM) or PBS, in addition to angiogenin (R&D Systems, Inc. Minneapolis, MN), or VEGF (Protein Laboratories, Rehovot, Israel) (1 μg/ml each). After 8 h of incubation, at 37°C, the plates were photographed, and the extent of tube formation was counted using Image J (NIH, Bethesda, MD) software. Five individual determinations were performed for each treatment.

### Immunofluorescence

For trT2-50 and actin co-staining in HUVECs, cells were cultured on PBS-covered slides in 12-well plates with 0.1% pork gelatin (Sigma-Aldrich). Cells were fixed with 3% Paraformaldehyde (PFA) (Merck Millipore, Dermstadt, Germany) containing 0.5% triton, washed three times with PBS and blocked with 5% donkey serum (Jackson ImmunoResearch). Rabbit anti-trT2-50 or mouse anti-hRNASET2 (Sigma-Aldrich) or rabbit anti-angiogenin (Merck Millipore) were added (1:100 dilution; prepared in 5% donkey serum) and incubated overnight at 4°C. After washing three times with TBST, the slides were incubated for 1 h with Alexa 488-conjugated anti- rabbit or anti- mouse antibodies (Invitrogen Life Technologies) or Rhodamine Red™-X (RRX)-conjugated anti- rabbit antibody (Jackson ImmunoResearch) and phalloidin tetramethylrhodamine B isothiocyanate–conjugated anti-rabbit antibody (Sigma-Aldrich). Then, the slides were washed and mounted with a mixture containing 30% mounting medium, 4′,6-diamidino-2- phenylindole (DAPI) (Santa Cruz Biotechnology Inc., Santa Cruz, CA) and 70% fluoromount (Sigma-Aldrich). The slides were viewed under a Leicactr4000 laser scanning confocal microscope.

### Colony-formation assay

This assay was performed essentially as described previously [6] with minor modifications. HT-29 cells obtained from the ATCC^®^ (Manassas, VA. Lot number 4487730) were derived from colon epithelial cells. Cells (10^5^ cells per 50-ml flask) were grown in 7 ml DMEM supplemented with 10% FCS, 1% glutamine, 1% antibiotic-antimycotic solution (Biological Industries, Bet Haemek, Israel) and in the presence or absence of either 1 μM trT2-50, mock (equivalent amount in PBS) or control (PBS). The cells were incubated at 37°C in a humidified atmosphere containing 5% CO_2_ and the growth medium was changed 24 h after cell implantation. After another 24 h, cells were seeded in 96-well plates (1 × 10^3^ cells/well/200 μl medium) in the presence or absence of 1 μM trT2-50, mock (equivalent amount in PBS) or PBS. After 5 days, the cells were fixed in 5% formaldehyde, 60% ethanol, and 5% acetic acid in water and stained with methylene blue. In each treatment, colonies that contained at least 100 cells were counted. Six individual determinations were performed for each treatment.

### *In vivo* assays

#### Xenograft model

All animal experiments were approved by the Ethics Committee for Animal Experimentation, Robert H. Smith Faculty of Agriculture, Food and Environment, the Hebrew University of Jerusalem, Israel.

The anticancer and antiangiogenic effects of trT2-50 were tested in an *in vivo* in a xenograft model. Viable HT-29 cells were injected subcutaneously (0.5 × 10^6^ cells/100 μl per mouse) into the left hip of female athymic Nude/nu mice, 5-6 weeks (Harlan, Rehovot, Israel). When tumor diameter reached 5-7 mm, 100 μl of trT2-50 (5 mg/kg) in PBS, mock (equivalent amount in PBS) or 100 μl PBS were injected into the tail vein, every other day, to a total of nine injections. Food was provided *ad libitum* under pathogen-free conditions. Tumors were measured with Vernier calipers twice a week. Tumor size (mm^3^) was evaluated by the formula 1/2LW^2^, in which L is the largest dimension and W is the dimension perpendicular to L [30]. The results are presented as mean relative tumor volume (RTV) = Vi/V_0_, where Vi is the tumor volume at any given time and V_0_ is that at the time of initiation of the treatment [31]. The experiments were terminated 21 days after the initial treatment. Mice were sacrificed and tumors were fixed with 4% formaldehyde in PBS, dehydrated by passing through serial ethanol concentrations and embedded in paraffin. Cross-sections (6-7 μm) were stained with H&E and examined by light microscope (BX-40, Olympus, Hamburg, Germany). Five mice were used for each treatment.

### Blood vessel analysis

In each median tumor cross section, the blood vessels were counted, and their areas were analyzed with Image J (NIH, Bethesda, MD) software as previously described [32]. A binary image was created using a threshold value midway between background (white) and blood vessels (black). The number and size of all black objects (blood vessels) greater than 10 pixels were determined using the particle analysis function of Image J. From these data, we determined total vessel area and the relative area (the ratio between total blood-vessel area and tumor-section area). From each tumor section, 2-4 different fields were assessed.

### Statistical analysis

Means were compared by analysis of variance (ANOVA). Differences were considered statistically significant at *p* < 0.01.

## Abbreviations

HUVEC: human umbilical vein endothelial cell
RNASE: ribonuclease
EK: enterokinase
IPTG: isopropyl β-D-thiogalactopyranoside
EDTA: ethylenediaminetetraacetic acid
SAB: sample application buffer
TBS: tris buffer saline
TBST: tris buffer saline with tween-20
RTV: relative tumor volume
H&E: hematoxylin and eosin
SDS-PAGE: sodium dodecyl sulfate polyacrylamide gel electrophoresis
DMEM: Dulbecco's Modified Eagle's Medium
FCS: fetal calf serum
FBS: fetal bovine serum
PBS: phosphate buffered saline
EDC: 1-[3-(dimethylamino)-propyl]-3-ethyl-carboimide methiodide
